# Benefits and perspectives on the use of biofuels

**DOI:** 10.1111/1751-7915.12356

**Published:** 2016-04-26

**Authors:** Juan‐Luis Ramos, Miguel Valdivia, Francisco García‐Lorente, Ana Segura

**Affiliations:** ^1^ Abengoa ResearchC/Energía Solar, 1 Palmas AltasSeville41014Spain

## Abstract

The current primary obstacle to biofuels is the current low price of fossil fuels, and the primary incentive to 2G biofuels is the growing world population and need to increase food suplies. Both of these will be increasingly subject to political, regulatory and legislative changes that will be positive for 2G biofuels.

Petrol prices between December 2015 and January 2016 have been at their lowest in years. These more affordable gasoline prices encourage less efficient car‐based transportation and longer trips, which is, in turn, expected to lead to increased carbon dioxide emissions and increased greenhouse gas levels in the atmosphere. Terrestrial transport can represent up to 20% of all C emissions to the atmosphere (www.eea.europa.eu). In addition to emissions from terrestrial vehicles, about 2% of all human carbon dioxide emissions are the result of aircraft emissions – many of which represent vacation trips. The relationship between tourism, as a global industry, and energy use is often neglected (Becken, 2002). This is despite the fact that if tourism continues to grow at currently predicted rates, it will lead to considerable increases in aircraft emissions by 2050. At present, the Natural Resources Defense Council indicates that it is estimated that air travel emits more than 650 million metric tonnes of carbon dioxide annually – equivalent to the pollution from 136 million cars, making the increased use of sustainable biofuels critical for reducing its carbon footprint (Martínez, 2014).

Kyoto Protocols and the most recent Paris Protocols are calling for the use of clean, green and renewable transportation fuels to replace gasoline, diesel and jet fuel (United Nations, 2016). Biofuels for motor vehicles are considered a potential alternative for carbon emission savings because biofuels are produced through processes that significantly reduce net emissions (Fargioni *et al*., 2008). A number of biofuel programmes have been implemented in the United States and the European Union with the aim of not only reducing emissions but also reducing the importation of fossil fuels and enhancing the security of fuel supply at a national level. Despite of these efforts, current estimates indicate that only about 1% of the energy used globally can be traced back to a biofuel source; therefore, there exists great opportunities to increase the use of renewable liquid fuels (Hill *et al*., 2006).

## First‐ and second‐generation ethanol

Currently, bioethanol is the most common biofuel. Almost 99% of it is produced from corn grain (and other cereals) and it is referred to as ‘first generation’ (1G) bioethanol. In the United States, ethanol production rates are in the range of 14–15 billion gallons per year at corn dry mills; these mills produce not only ethanol but also corn oil and dry distillers’ grains (DDG) – a product that is used as animal feed (Mosier and Ileleji, 2014). It is estimated that the ethanol produced in the United States serves to replace about 500 million barrels of petroleum annually. Europe currently produces about 2.5 billion gallons of ethanol per year at around 70 ethanol production plants (Voegele, 2013). In Brazil, which is the second largest producer and user of ethanol, about 6.2 billion gallons of ethanol were produced in 2014 from sugarcane, and this ethanol also belongs in the 1G category (Barros, 2014).

Although 1G bioethanol is recognized as a renewable energy source, its production is not free from controversy, as it has been the subject of a wide range of societal and political debates. Questions about costs, security of energy supply, greenhouse gas emissions, sustainability of production systems, impact on food production and on biodiversity are some of the many issues which have been raised regarding this source of energy.

Hill *et al*. (2006) proposed that for a biofuel to be a viable gasoline alternative, it should provide a net energy gain, have environmental benefits, be economically competitive and be produced in large amounts without reducing food supplies. It is clear that 1G bioethanol does not fulfil all these requirements, although many process modifications have been made in the last years to improve sustainability. Continuous improvements to 1G bioethanol technology have led to savings of around 3.7 pounds of CO_2_ per every gallon of ethanol produced in standard 1G plants – savings that are achieved through the capture and liquefaction of CO_2_ during fermentation. Although volatility of corn grain and ethanol is high, a number of studies suggest that every $1 spent to produce ethanol through input feedstock yields $1.83 in finished products, that is, alcohol, corn oil, CO_2_ and DDG, which guarantees ethanol's economic viability.

In an attempt to address the food versus fuel controversy, the biofuel industry has searched for new, alternative feedstock sources for biofuel production. This approach is necessary for other reasons, namely because it has been calculated that even if all the US corn was used to produce biofuels, this would only satisfy 12% of the demand for gasoline (Hill *et al*., 2006). The use of non‐food sources, such as corn stover, wheat straw, woody biomass or organic matter from municipal solid waste (MSW), for ethanol production is known as ‘Second‐generation’ (2G) bioethanol. 2G ethanol has been considered to be a promising alternative to 1G ethanol. These 2G biofuels are considered to be more energy efficient than conventional fossil fuels and are more environmentally friendly as well. While the number of 1G facilities worldwide is not increasing (Voegele, 2013), many initiatives have been put in place to increase 2G plants. Several companies have recently announced the opening of commercial cellulosic ethanol facilities, namely INEOS‐bio, POET‐DSM, Dupont and Abengoa, although a number of hurdles have been encountered that have delayed launch dates and prevented steady production rates, such that they are running at a small fraction of their nameplate capacity. The US cellulosic ethanol capacity at the end of 2015 was estimated at 86 million gallons; however, only 1.6 million gallons were registered. In retrospect, this may not be fully unexpected given the novelty of this incipient industrial process. The United States has put in place a number of initiatives to promote 2G biofuels. At the federal level, the Renewable Fuel Standard (RFS2) mandates increasing the volume of biofuels to be blended into gasoline and diesel, while providing a premium price for biofuel based on a credit system known as ‘RINs’ (for more information on the current status of the RFS2 can be found at ‘EPA cuts US biofuels quota through 2016’). At the state level, California's Low Carbon Fuel Standard (LCFS) mandates additional amounts of low carbon fuels to be blended into petroleum products above and beyond the RFS2. The LCFS also has credits attached to each gallon of biofuel, for which their monetary value is determined based on the carbon intensity of the fuel.

The production of 2G bioethanol usually requires three major steps: a physicochemical pre‐treatment, an enzymatic breakdown of biomass into its constituent sugars and fermentation (Taherzadeh and Karimi, 2007; Álvarez *et al*., 2016). At present, the main hurdles in 2G ethanol seem to arise from mechanical issues in the handling of materials and the efficient operation of the pre‐treatment units. Pre‐treatment is required to make the polysaccharides (cellulose and hemicellulose) in lignocellulosic material accessible. The process depends upon cellulases and hemicellulases that convert complex sugars into simple sugars, which can then be fermented (Taherzadeh and Karimi, 2007). Although a number of studies point to the use of ionic liquids as a new potential pre‐treatment (Uppungundla *et al*., 2014; Ding *et al*., 2016), their current prices make them non‐competitive at the industrial scale. Because of this, dilute acid or caustic treatments followed by steam treatments are the most commonly used.

It is well known that in nature a number of fungi secrete a set of enzymes that allow them to grow by metabolizing lignocellulosic residues (Wackett, 2011; Zhou *et al*., 2015). The use of fungi to produce enzymatic cocktails has been iteratively improved and a series of recombinant strains to produce these cocktails are available. The ultimate goal of using these cocktails on biomass is to enable the release > 80% of the sugars present in celluloses and hemicelluloses as monosaccharides. 2G enzymatic cocktails have been commercialized by Novozymes, Genencor and Abengoa Biotec, among others. Enzyme manufacturing for 2G has been achieved in 400 m^3^ fermenters – a process that has been shown to be safe, efficient and profitable, yielding 100 g of protein per kg of cocktail (Abengoa's own source). In tandem, enzyme efficiency has been increased by a factor of 10 by Abengoa.

Fermentation of sugars released from corn stover, sugarcane and other agricultural residues requires specialized yeasts able to simultaneously ferment glucose and xylose (Heer and Sauer, 2008). The yeasts used in 2G fermentation are genetically modified to convert more than 96% of glucose and more than 90% of xylose into ethanol with overall fermentation yields >90% of the theoretical maximum – results that demonstrate how far this part of the process has come.

Despite these gains, at present 2G biofuels are not cost‐competitive versus 1G biofuels (Somerville *et al*., 2010). Investment in the construction of the 2G plants, feedstock prices and operational costs associated with enzymatic hydrolysis comprise a large fraction of the costs of producing 2G biofuels.

Thus, the reduction of capital costs is a key factor for 2G ethanol affordability – one that will require a very significant ‘learning curve’. Costs associated with setting up 2G plants will likely only diminish after several plants have been built; however, even if high subsidies favour the construction of several full‐size plants in the next few years, the lessons learned will likely not significantly reduce the costs of 2G biofuels for years (Abengoa's own source). Therefore, it is expected that even in 10 years’ time, 2G biofuels may still be more costly than 1G biofuels.

The second key point to address before 2G bioethanol can become economically feasible is the reduction of operational costs – an issue that has been cited in several recent techno‐economic studies (Macrelli *et al*., 2012; Gnasounou and Dauriat, 2010). Most of these costs rest in the raw material used to feed the process, as well as in the cost of enzymes for enzymatic hydrolysis. Feedstock prices are governed by supply and demand market forces, as well as supply chain logistics (Somerville *et al*., 2010). Although biofuel developers have been willing to pay for corn stover and other residues, farmers have been cautious about removing stover and straw from their fields because it is known that they help to fertilize the land and protect it against weather changes. Farmers are also unsure of the long‐term stability of 2G biofuels and are cautious of devoting specific resources towards collecting residues or developing a supply chain when there may be no further cellulosic biofuel plants built. At present, 2G plants pay above what they expected for feedstock and may need to actively participate in the creation of more affordable supply chains. This means recruiting interested farmers, developing the right machinery for residue collection, hiring people to collect and deliver the feedstock, and then, developing safe ways of storing and handling it. These problems have been well known for years, but are proving to be very challenging. To address this, the industry is currently evaluating various alternatives to the existing feedstock supply chain. One example of this is the use of an intermediate storage or processing at depot (Lamers *et al*., 2015) – an approach that successfully reduced the risk of raw material availability shortfalls. Despite this success, further supply chain innovations are needed to impact the cost‐competitiveness of 2G bioethanol. An early estimation of the price of corn stover delivered to the ethanol plant gate was in the range of $25–50/tonne. DuPont, who has done significant work in feedstock supply chain development, estimated for 2015 a cost of $55/tonne, although prices as high as $75/tonne have been quoted. In Europe, agricultural residues may be purchased at a cost below $50/tonne.

While enzyme costs in 1G ethanol are not significant to the total alcohol production cost, in 2G technology the enzyme cost can be in the range of 15% of total ethanol operation costs. Thus, current efforts in the 2G enzyme technology are directed towards enhancing the efficiency of enzyme production and enhancing the activity of these enzymes. At present, Abengoa's 2G enzymatic cocktail costs are estimated at around 0.4–0.5 US$/gallon ethanol.

## Other feedstocks

Although most 2G bioethanol production efforts have been focused on the use of corn stover and sugar cane straw, other agricultural residues like wheat straw, sorghum straw, woody biomass and sugarcane bagasse are also being considered. They were predicted to have stable prices and could provide options for long‐term fixed price off‐takes (Somerville *et al*., 2010). Other less conventional agricultural residues also have promise. For example, Corbin *et al*. (2015) published in *Bioresource Technology* that up to 400 l of bioethanol could be produced through the fermentation of 1 tonne of grape marc (the leftover skins, stalks and seeds from winemaking). Other potential biomass starting materials for 2G bioethanol production are vegetables that, either at the place of production or at market, are removed from the supply chain and are not sold to the public. New approaches to deal with the set of different potential feedstocks and the use of more than one feedstock at the same time deserve more study.

Forest wood resources are some of the highest potential non‐food biofuel feedstocks in terms of availability, and this availability has started to attract global attention. Felipe Benjumea, former President of Abengoa, foresaw the benefits of harnessing fast‐growing trees because they provide perennial renewable feedstocks, which would not compete with foods and could be more sustainably harvested. Along this line of thinking, researchers at several institutions have shown the outstanding diversity and adaptability that make trees a global renewable resource of fibre for ethanol production (Myburg *et al*., 2014). Of various forest woods, willow trees have demonstrated a higher potential for use in biofuel production, because they produce large quantities of accessible sugar, are fast‐growing and can tolerate harsh environmental conditions, such as windy slopes and poor soils. Researchers at Imperial College London, in collaboration with Rothamsted Research, explored why growing willow trees at an angle improved their biofuel yields. The researchers found that growing the willow trees at a 45° angle resulted in plants producing up to five times more sugar than plants grown normally (Brereton *et al*., 2015). This increase was found to correlate with substantial xylem tissue remodelling involved in wood fermentation, but the molecular basis of why and how this happen remains unexplored.

As in the 2G process with herbaceous residues, the main hurdle in the use of woody biomass for 2G biofuels resides in the price of wood and supply chain costs. In addition, enzyme costs are expected to be higher than with herbaceous straw wastes due to the intricate bonds of lignins and polysaccharides in woody mass (Álvarez *et al*., 2016), and because the hydrolysis of woody biomass leads to the production of a number of chemicals (i.e. acetic acid and aromatic compounds) that act as feedback inhibitors of the enzymes (Reviewed by Álvarez *et al*., 2016) or interfere with the fermentation of sugars (Heer and Sauer, 2008).

It is estimated that advanced biofuels from MSWs and other residues could replace 16% of fuel used by the U.S. transportation sector by 2030. A study by Kalago *et al*. (2007) stressed the importance of ensuring that MSWs are sustainably sourced, and that if they are, their use could reduce related greenhouse gas emissions savings by 65%, even when taking into account all possible indirect emissions. The organic fraction of MSW is around 61% in the United States and, according to the EPA, if the 164 million tonnes that are currently diverted to landfills in the United States were converted to bioethanol, about 7.5 billion gallons of ethanol would be produced from biowaste, representing savings of around 250 million barrels of petrol.

## Ethanol, isobutanol and n‐butanol blends in gasoline: symbiont biofuels

In 1944, Charles Kettering identified ethanol as a blending agent and estimated an optimal blend to be 30% ethanol in gasoline (Kettering, 1944). In the United States, by law, ethanol can be blended with gasoline up to a 10%. This gasoline is known as E10 – fuel that requires no major technological adjustments to existing infrastructure or vehicle motors. Higher blends (i.e. 15% ethanol in gasoline) require small modifications to vehicles and derogation of hydrocarbon emissions limits. Adding 10% ethanol in gasoline reduces the emission of fine particulate matter by 36%, and by as much as 65% in cars with large displacement volumes. Benzene is often identified as the most important toxic and carcinogenic compound found in car exhaust, and E10 gasoline emitted 25% less benzene (Niven, 2005).

The blending of gasoline with medium chain alcohols such as butanol has been authorized by the American Society for Testing and Materials. Two of the butanol isomers, isobutanol and *n*‐butanol, are considered useful biofuels (Coons, 2012). Currently, debates within the biofuel field are positioning butanol a better fuel component than ethanol. Those who support butanol point to three key benefits: (i) butanol has a higher fuel density; (ii) it can be added to gasoline at a higher blend ratio of 1.6:1 (i.e. E10 is equivalent to B16); and (iii) it is highly compatible with existing petroleum distribution systems (Green, 2011), including fuel pumps. However, butanol has also some disadvantages in comparison to ethanol:butanol has a lower octane rating, a lower heat of evaporation and a significantly lower Reid vapour pressure (RVP) (Wu *et al*., 2014).

Current biological production of butanol is very limited; only one company (Gevo, Luverne, MN, USA) has reported the bioproduction of isobutanol, with a total yield of 50 000 gallons in 2014. *n*‐butanol and isobutanol can also be produced from lignocellulosic materials (Ding *et al*., 2016) and from corn grain (Green, 2011). A detailed techno‐economic analysis of cellulosic isobutanol, *n*‐butanol and ethanol has been carried out by Tao *et al*. (2014); they concluded that the relative energy returned on investment for each biofuel is isobutanol < ethanol < *n*‐butanol. It has been proposed that butanol and ethanol can be blended together to make a better fuel blending agent (Elfasakhany, 2015). A combined alcohol blend of about 18% ethanol and 12% butanol can maintain the original Blendstock for Oxygenated Blending RVP, but would lack optimal latent heat of evaporation (Brandon and Ezike, 2015; Elfasakhany, 2015).

## Conclusions and further perspectives

The value of biofuels goes beyond their use as transportation fuels, and attention should be given to the economic and environmental benefits of the co‐products of biofuels. Both 1G and 2G biofuel industries serve to significantly reduce greenhouse gas emissions and our reliance on crude oil, encouraging energy diversity, while promoting the creation of a large number of rural jobs. Today, with the blending limit in the United States set at 10% and gasoline prices going down, corn ethanol producers have relatively little incentive to partner with cellulosic‐based fuel companies as they regard next‐generation ethanol as a market competitor that would only displace their existing corn ethanol. Presently, the long‐term success of 2G ethanol requires financial incentives and supportive regulations, which are instrumental for driving the commercial production and adoption of advanced biofuels.

In looking towards 2030, there is great potential for the production of biofuels from non‐edible plant materials and MSW residues. In addition to ethanol, the production of other chemicals, such as butanol farnesene and several carboxylic acids (Eggleston and Lima, 2015; Ding *et al*., 2016; Ramos *et al*., 2016), from non‐food feedstocks hold great potential for increasing the value and usefulness of biofuels.
